# Consenting patients for elective procedures during the pandemic: Are we consenting for risk of nosocomial COVID-19 infection

**DOI:** 10.1177/17504589211045235

**Published:** 2021-11-10

**Authors:** CU Menakaya, M Durand-Hill, O Okereke, DM Eastwood

**Affiliations:** The Catterall Unit, Royal National Orthopaedic Hospital, Stanmore, UK

**Keywords:** COVID-19, Consenting, Surgical procedures, Pandemic, Perioperative care

## Abstract

**Introduction:** Nosocomial COVID-19 increases morbidity and mortality in patients undergoing surgical procedures. This study assesses the consenting process in patients admitted for surgical procedures with regard to risks of contracting nosocomial COVID-19 infection during the three lockdown periods in the United Kingdom.

**Methods: **Retrospective review of consecutive surgical patients admitted to our tertiary referral centre for surgical procedures during the lockdown periods in the United Kingdom. Data from our hospital’s electronic theatre database cross-referenced with the online surgical operative, admission and discharge records were reviewed by three independent reviewers.

**Discussion:** A total of 180 patients (104 males and 76 females) were studied. No patients tested positive perioperatively for COVID-19. The first lockdown had a significantly larger proportion of consultants consenting (P < 0.001). Surgeons consented patients for risk of COVID-19 infection in 34.4% of cases, COVID-19-related illness in 33.9%, inpatient Intensive Care Unit (ITU) admission secondary to COVID-19 infection and risk of death due to COVID-19 in 0.0% and risk of death secondary to inpatient COVID infection in 1.1%.

**Conclusion:** As surgical activity continues and COVID-19 persists, surgeons should be vigilant and ensure proper documentation for consent regarding COVID-19-related complications in line with the Royal College of Surgeons of England guidelines.

**Provenance and Peer review:** Unsolicited contribution; Peer reviewed; Accepted for publication 22 August 2021.

## Introduction

The COVID-19 pandemic continues to pose a severe health crisis with increased morbidity and mortality worldwide (Wang et al 2019, Zhu et al 2019). Despite the public health focus on curbing the pandemic and restricting the spread of new variants, the number of patients on the elective surgical waiting list continues to rise and cannot be ignored. Outcomes from surgery have been reported to be poor if a patient has COVID-19 or contracts COVID-19 during their hospital admission or attendance (RCS 2020). Hospitals have instigated robust COVID-19 infection prevention pathways to reduce this risk but despite these precautions, studies have reported incidences of nosocomial COVID-19 infections in admitted patients ranging from 12.5% to 41% (Carter et al 2020, Wang et al 2019, Wee et al 2020, Wong et al 2019, Yu et al 2020). Mortality rates up to 21% in patients undergoing elective surgery who were diagnosed with COVID-19 perioperatively have also been reported (COVIDSurg Collaborative 2020, Lei et al 2020, NELA Project Team 2019, Wu et al 2019). All mortalities are attributed to respiratory failure.

Therefore, all patients admitted for surgical procedures during this pandemic must be informed of the risk of nosocomial COVID-19 infection and the potential effects on their perioperative care. This is reinforced by the Montgomery vs Lanarkshire Health Board (2015) UKSC 11case, the Royal College of Surgeons, and surgical associations which direct doctors to take all reasonable care to ensure that patients are aware of all potential risks involved in their recommended treatment (BASS 2020, ESSKA 2020, Guidance I 2019). Also, the consenting process should contain full documentation that these risks were discussed with the patient (BASS 2020, Consent to treatment 2020, ESSKA 2020, Guidance I 2019, Sokol & Dattani 2020). We believe that the failure of doctors to inform patients of the perioperative risks of nosocomial COVID-19 infection falls short of best practice (Sokol & Dattani 2020). Surgeons must be made aware of the need to discuss these risks with their patients.

This audit was designed to assess the consenting process during the three lockdowns in the United Kingdom in patients admitted to a tertiary elective centre for surgical procedures with regard to risks of contracting nosocomial COVID-19 infection.

## Methods

We retrospectively reviewed consecutive patient cohorts admitted to our elective tertiary referral centre for surgical procedures during the lockdown periods in the United Kingdom (23 March to 23 April 2020, 5 November to 2 December 2020 and 4 January to 4 February 2021).

Data were collected from our hospital’s electronic theatre database and medical records and cross-referenced with the online surgical operative, admission and discharge records.

Patients admitted for surgical procedures were assessed for baseline demographics, pre-surgery comorbidities, type of surgery, American Society of Anaesthesiologists (ASA) grade, associated medical comorbidity, length of hospital stay, pre-surgical COVID-19 status, development of post-surgical COVID-19 infection and the number of post-surgical COVID-19-related deaths.

To understand the assessment of nosocomial surgical COVID-19 risk, three independent reviewers assessed all consent forms for major surgical procedures undertaken in our institution during the three study periods for information provided by the person taking consent on surgical-related COVID-19 risks as documented on the consent form. These risks were subdivided into the general risk of COVID-19 infection, risk of COVID-19-related illness, risk of COVID-19-related inpatient ITU admission, risk of COVID-19-related deaths and risk of death due to inpatient COVID-19 infection.

### Statistical Analysis

All data were collated in Microsoft Excel. Statistical analysis was conducted using SPSS version 18.0 (SPSS Inc., Chicago, IL, USA). Continuous variables are presented as the mean, standard deviation and range. Categorical variables are reported as frequency and percentage. Parametric Student t-test and nonparametric Mann–Whitney U-test were used on continuous dependent variables. Pearson’s chi-squared test was used on categorical variables. A two-tailed p-value of <0.05 was considered significant.

## Results

A total of 180 patients (104 males and 76 females) were included in our sample. Patient demographics for our study population stratified by sample month are shown in Table 1. No patients tested positive preoperatively or postoperatively for COVID-19. The grade of consenter stratified by sample month is presented in Table 2; March 2020 had a significantly larger proportion of consultants consenting than October 2020 or January 2021 (P < 0.001). Documentation of risks stratified by sample month is presented in Table 3.

**Table 1:** Patient demographics

	March 2020 (%)	October 2020 (%)	Jan 2021 (%)
Age	51.1 (S.D 24.0)	53.5 (S.D 20.0)	55.6 (S.D 24.1)
Length of stay	14.0 (S.D 49.5)	4.6 (S.D 6.5)	17.0 (S.D 56.1)
Gender			
Male	43 (63.2)	36 (60.0)	25 (48.1)
Female	25 (36.8**)**	24 (40.0)	27 (51.9)
ASA grade			
1	18 (26.5)	20 (33.3)	9 (17.3)
2	32 (47.1)	28 (46.7)	24 (46.2)
3	15 (22.1)	11 (18.3)	19 (36.5)
4	3 (4.4)	1 (1.7)	0 (0.0)
–ve Preoperative COVID test	68 (100.0)	60 (100.0)	52 (100.0)
–ve Postoperative COVID test	68 (100.0)	60 (100.0)	52 (100.0)
Postop complications	5 (7.9)	2 (3.3)	8 (15.4)
7-Day mortality	0 (0.0)	0 (0.0)	0 (0.0)
30-Day mortality	2 (2.9)	0 (0.0)	0 (0.0)

**Table 2:** Grade of consenter by month

Grade of consenter	March 2020 (%)	October 2020 (%)	Jan 2021 (%)
Consultant	64 (94.1)	25 (41.7)	16 (30.8)
Fellow	2 (2.9)	10 (16.7)	13 (25.0)
Registrar	0 (0.0)	23 (38.3)	19 (36.5)
SHO	0 (0.0)	2 (3.3)	4 (7.7)

SHO: Senior House Officers.

**Table 3:** Documentation of risks by month

Documentation	March 2020 (%)	October 2020 (%)	Jan 2021 (%)	P-value
Risk of COVID infection	1 (1.5)	39 (65.0)	22 (42.3)	<0.001
Risk of COVID-related illness	0 (0.0)	39 (65.0)	22 (42.3)	<0.001
Risk of inpatient ITU admission due to COVID	0 (0.0)	0 (0.0)	0 (0.0)	–
Risk of death due to COVID	0 (0.0)	0 (0.0)	0 (0.0)	–
Risk of death 2nd to inpatient COVID infection	0 (0.0)	0 (0.0)	2 (3.8)	0.083

Two patients died within 30 days of their operation. Both cases were in patients who were ASA grade 3 or greater. One patient was a right hemiarthroplasty for an intracapsular neck of the femur, the other was a dynamic hip screw for an extracapsular hip fracture. Neither had consented with respect to COVID-19 specific complications. Both had in-hospital complications postoperatively; one patient developed a urinary tract infection (UTI) and a pulmonary embolism (PE), the other developed postoperative delirium.

Senior House Officers (SHO) were the best performers with regard to the documentation of the risk of COVID-19 infection (P = 0.022), risk of COVID-19-related illness (P = 0.022) and risk of death secondary to inpatient COVID-19 infection (P = 0.002). Consultants within our sample were better than registrars at documenting the risk of COVID-19 infection and COVID-19-related illness on consent forms (Table 4).

**Table 4:** Documentation of risks during October 2020 and Jan 2021 by grade

Documentation	Consultant	Registrar	SHO	P-value
Risk of COVID infection				0.022
No	16	35	0	
Yes	25	30	6	
Risk of COVID-related illness				
No	16	35	0	0.022
Yes	25	30	6	
Risk of inpatient ITU admission due to COVID				–
No	41	65	6	
Yes	0	0	0	
Risk of death due to COVID				–
No	41	65	6	
Yes	0	0	0	
Risk of death 2nd to inpatient COVID infection				0.002
No	41	65	4	
Yes	0	0	2	

SHO: Senior House Officers.

## Discussion

We report the results of our audit into consenting practices within our institution, during the three COVID-19 lockdowns within the UK. Consultant surgeons consented patients for risk of COVID-19 infection 34.4% of the time, COVID-19-related illness 33.9% of the time, inpatient ITU admission secondary to COVID-19 infection 0.0% of the time, risk of death due to COVID-19 0.0% of the time and risk of death secondary to inpatient COVID-19 infection 1.1% of the time.

Other studies have demonstrated similar rates of COVID-19-related risk documentation (21.5%) on consent forms. This is far below guidance from the Royal College of Surgeons and British Orthopaedic Association on consent, which states that patients who are undergoing operative procedures during the pandemic should be informed of (1) the risk of contracting COVID-19 whilst in hospital, (2) the risk of surgery for patients who have tested positive for COVID-19, and (3) changes in the coordination of care due to pandemic response and a possibility of the scarcity of resources, for example, for postoperative support and rehabilitation.

The exact risk to patients undergoing orthopaedic surgery who develop COVID-19 is not fully understood. As a result, providing informed consent to patients undergoing surgery during the pandemic periods covered by this audit was a challenging endeavour. Clough et al (2020) estimated that the rate of COVID-19 infection following orthopaedic trauma surgery was 0.9% at elective sites and 6.5–8.3% at mixed sites. Furthermore, they reported that the COVID-19 mortality rate in patients that developed COVID-19 perioperatively was between 50% and 71%.

Within our site, no patients developed COVID-19 perioperatively. One possible explanation for our lower rate of perioperative COVID-19 was that for the second two audit periods, all hospital staff underwent Ploymerase chain reaction (PCR) tests every two weeks, supplemented in the last period by four lateral flow tests within each two-week period. This differed from the staff at Clough et al’s (2020) elective site who are tested only when symptomatic. Furthermore, staff members did not work across elective and mixed sites, and thus were less likely to cross-contaminate our clean elective surgical site.

SHOs were the best performers with regard to documenting risks of COVID-19 infection or related illness (P = 0.022) and risk of death secondary to COVID-19 (P < 0.001). One possible explanation for this is that SHOs do less consenting than registrars and consultants within our institution and therefore may be less complacent about the process. Furthermore, SHOs occasionally perform consenting whilst supervised and therefore there are two healthcare individuals present for the consent process avoiding error. Lastly, as few SHOs consent within our institution, our sample of SHO consent forms may not be a true representation of the process.

“We found a significant difference between the rate of documentation of COVID-19-related complications and month (P < 0.001), with March 2020 (the first UK lockdown period having the lowest rate followed by January 2021 and then October 2020.” The low levels of documentation in March are not surprising given that the first paper published on perioperative complications during the pandemic was published in April 2020 (Clough et al 2020).

Our study has several limitations, our sample size is small and from one institution. Therefore, rates reported of consenting for COVID-19-related complications during trauma and orthopaedic surgery may not be comparable to other institutions/across the UK. However, it highlights a need to stay vigilant and ensure proper documentation of consent regarding COVID-19-related complications.

## Conclusion

The findings of this study suggest that only 34.4% of patients were informed of the risk of developing COVID-19 perioperatively and the associated problems. As elective activity resumes, surgeons should be vigilant and ensure proper documentation for consent regarding COVID-19-related complications in line with the Royal College of Surgeons of England guidelines.

*No competing interests declared*
